# A novel approach to noninvasive monitoring of dissolved carbon dioxide in small-scale cell culture processes

**DOI:** 10.3389/fbioe.2022.968294

**Published:** 2022-09-06

**Authors:** Vida Rahmatnejad, Michael Tolosa, Xudong Ge, Govind Rao

**Affiliations:** Center for Advanced Sensor Technology, Department of Chemical, Biochemical and Environmental Engineering, University of Maryland, Baltimore County, Baltimore, MD, United States

**Keywords:** noninvasive, rate-based, sensor, dissolved CO2 (DCO2), dissolved O2 (DO), mammalian cell culture

## Abstract

Disposable small-scale vessels are commonly used in cell culture studies in academia as well as early stages of bioprocess development. These types of research are crucial for our understanding about cells and bioprocesses as they provide important information regarding different parameters affecting cells. Dissolved carbon dioxide (DCO_2_) is one main parameter affecting cell metabolism. It is also an indicator of cell culture well-being. Despite CO_2_ being a critical process parameter, there is a lack of appropriate monitoring system for CO_2_ in small-scale vessels. Here, we present a membrane-based noninvasive method for measuring DCO_2_ in cell culture medium. The idea was achieved by modifying a T-flask and replacing a small area of it with CO_2_ permeable silicone membrane. In the proposed method, the concentration of CO_2_ dissolved in the cell culture medium is determined by measuring the initial diffusion rate of CO_2_ through a silicone membrane attached to the bottom wall of the T-flask. The measurement method was validated previously, and the efficacy of the noninvasive method was evaluated by growing *E.coli, Pichia pastoris*, and CHO cells in the proposed prototype. The results obtained from this method were verified with other quantitative data obtained from the process such as optical density (OD), cell density, dissolved oxygen (DO) and pH. The results show that the proposed membrane-based method is an effective way for completely noninvasive monitoring of DCO_2_ in small-scale cell culture processes. Additional diffusing species such as oxygen could also be measured using the same approach.

## 1 Introduction

The promising effectiveness of biopharmaceuticals, especially cell therapy products has led to a significant interest in cell culture in the past few decades (Abou-el-Enein et al., 2021; Eaker et al., 2013). Cell culture is the process of growing cells isolated from animal or plant tissues in laboratory and under aseptic conditions. To provide the optimal conditions for the cell culture process, important environmental parameters such as dissolved CO_2_ (DCO_2_), pH, and dissolved oxygen (DO) must be monitored throughout the process. Monitoring systems provide detailed information from the process. This information is critical for developing appropriate control systems and enhancing the productivity and efficiency of the process (Grilo and Mantalaris 2019; Lashkari 2017; Takahashi et al., 2017). CO_2_ is a nonpolar molecule capable of crossing the cell membrane and decreasing the intracellular pH. This pH change will affect the enzymes and consequently cellular metabolism. Several studies show the correlation between CO_2_ and other parameters such as lactate production, glycosylation, and product quality throughout the cell culture process (Pattison et al., 2000; Michio and Hossain 2018; Vohwinkel et al., 2011; Duarte et al., 2020). The physiological level of CO_2_ for mammalian cells is defined within the range of 4–7 kPa and increasing DCO_2_ level beyond this range has negative effect on the cell growth (Brunner et al., 2016). In fact, a decrease in cell proliferation is observed in the presence of high concentrations of CO_2_. Furthermore, high levels of CO_2_ in the beginning of the culture could result in a long lag phase. Protein glycosylation could also be impacted by high levels of CO_2_. In other words, elevated CO_2_ has inhibitory effect on the growth, and metabolic rate of cells. Therefore, studies show that product quality could be improved by controlling the CO_2_ level (Chopda, et al., 2020; Günter et al., 2018; Schmelzer and Miller 2002; Borys, et al., 1993; Gupta et al., 2013; Mowbray et al., 2021). The results from a comprehensive survey among more than 1,500 scientists indicate that more than 70% of researchers have not been able to reproduce experiments conducted by other scientists, and almost half of them have not been able to reproduce their own experiments (Baker 2016). Small-scale cell culture studies are crucial at early stages of biomanufacturing processes as they provide important information about different factors influencing the process. Yet these studies are not an exception in reproducibility crisis (Klein et al., 2021). By equipping small-scale cell culture vessels with monitoring systems, more quantitative data will be provided. This data is helpful in identifying the state of the culture, providing more reliable information from bioprocesses, developing appropriate control systems, controlling the culture conditions, and improving the products (Ahuja et al., 2015; Lashkari 2017; Dakhore, et al., 2018). Considering the importance of DCO_2_, several techniques are developed to measure this parameter. However, these monitoring methods are not appropriate for small-scale cell culture processes as they are associated with drawbacks such as being invasive, sizeable, and costly. Among available methods, optical sensors are the most suitable sensors because of having high sensitivity and being free of electromagnetic interferences. Despite these advantages, optical sensors are not widely used in academia as well as industry as there are still concerns about the adverse effects resulting from the direct contact of cells with patches. The effect of LED light on the cell culture medium is another concern related to optical sensors. Another disadvantage of the optical sensors is that for adherent cell lines, patches must be treated to enhance the cell anchorage to them, and this process is often tedious. Additionally, introduction of patch to the cell culture vessel can be a source of contamination, and this must be avoided in cell culture processes specifically for sensitive applications such as cell therapies. Durability of optical sensors is normally limited; therefore, they are not the best candidates for long-term cell culture processes (Ge et al., 2018; Holzberg et al., 2018). Other methods such as circulation direct monitoring and sampling system (CDMSS) were developed recently for monitoring DCO_2_. This method is applicable in both gas and liquid phases and works by placing a circulation bypass component in the site to sample. This component is tilted to avoid the cells from being accumulated in the circulation part. Despite the advantages of the system such as not interrupting the shaking of the culture during the sampling, the CDMSS clogging prevention is required when pellets form in the culture. Another disadvantage of this method is that the system is not appropriate for cell culture processes with low volume of medium such as cultures in T-flasks (Takahashi et al., 2017). To address most of the aforementioned issues, a rate-based method previously was reported by authors’ lab. The system works based on fully submerging a silicone sampling loop in the cell culture medium. Silicone is permeable to CO_2_ (Zhang and Cloud, 2006). Therefore, during the cell culture process, the CO_2_ in the cell culture medium diffuses through the silicone layer, and is collected in the sampling loop and circulated to the sensor for measurements (Chatterjee et al., 2014). The sensor is located outside of the cell culture environment, and is equipped with a microcontroller, a pump and two valves. The concentration of the dissolved CO_2_ is correlated to the initial diffusion rate of the CO_2_ through the permeable silicone sampling loop. Each cycle of CO_2_ measurement consists of two periods: purge period and recirculation period. In the start of measurement, N_2_ gas is purged into the sampling loop to remove any CO_2_ remaining in the loop. This step is the purge period. After completion of the purge period, the recirculation period starts during which CO_2_ diffusing through the silicone layer is collected in the sampling loop, and transferred to the CO_2_ sensor (K30 CO_2_ sensor from CO2Meter.com) using impermeable tubes (Chopda et al., 2020). The measurement method was validated by comparing the CO_2_ measurements from hybridoma culture *via* rate-based method with the measurements *via* the commercially available optical sensors. The results show that the rate-based method is an effective way to measure the CO_2_ in the cell culture medium (Chatterjee et al., 2014). Feedback from industry partners was positive. However, the need to introduce a sampling loop into the vessel was perceived as cumbersome because of the inevitable drawbacks associated with the design. First, the presence of the sampling loop in the cell culture medium increases the risk of contamination. Avoiding contamination is one of most important points in cell culture process, and it is specifically important in cell therapies where cells cannot be terminally sterilized because of their large size and being fragile (Gebo and Lau 2020). Second, to be able to measure the CO_2_ in the cell culture medium, the sampling loop must be fully submerged in the cell culture medium, which is a requirement that cannot be achieved in cell culture processes with low volumes of medium. Additionally, the presence of a sampling loop in the cell culture environment causes shear stress when shaking or mixing is applied during the process, and this could result in unwanted manipulations in the cell destiny such as promoting differentiation in stem cells (Maul et al., 2011; Dan et al., 2015; Huang et al., 2021). To address these issues, here we report an effective way to measure the CO_2_ dissolved in the cell culture medium without using a sampling loop. The approach to the noninvasive monitoring of DCO_2_ in T-flasks adopts the idea from a previous study by authors’ lab for noninvasive oxygen monitoring where an optical patch was mounted outside of the cell culture vessel (Gupta et al., 2013). In the proposed method, a diffusible surface is created in a T-flask by replacing a small area of the vessel with silicone membrane permeable to CO_2_. For CO_2_ measurement the sampling is conducted *via* the diffusible surface provided.

## 2 Materials and methods

### 2.1 Experimental setup

#### 2.1.1 T-flask preparation

**FIGURE 1:**
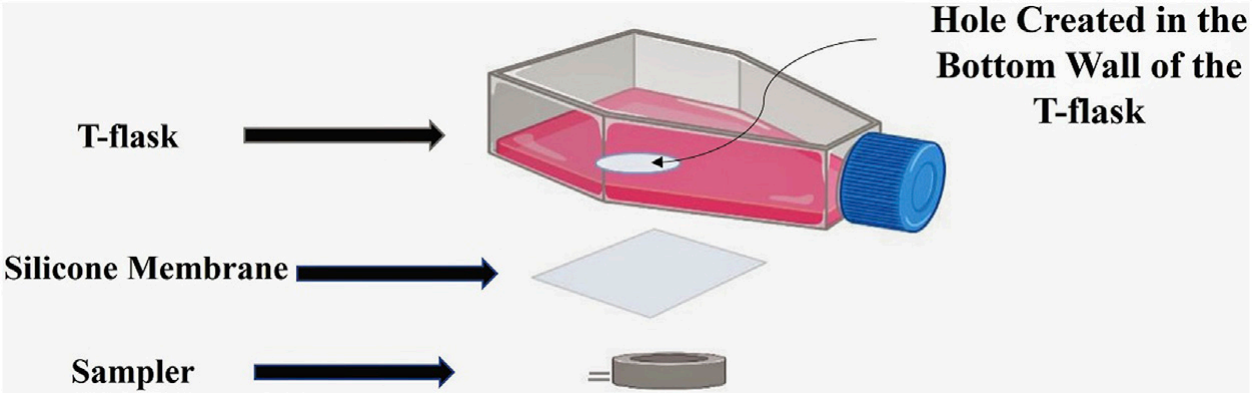
The proposed design for noninvasive CO_2_ measurement. A hole is first created in the bottom wall of the T-flask, and covered by a silicone membrane. A sampler is then attached on the silicone membrane from outside of the T-flask.

The prototype for noninvasive measurement of DCO_2_ was designed by creating a hole in the bottom wall of the T-flask and attaching a silicone membrane on the hole from outside of the T-flask. Silicone membrane is permeable to CO_2_ and facilitates the diffusion of CO_2_ during the cell culture process. A sampler is then attached on the silicone membrane from outside. When attaching the sampler on the silicone membrane, the cavity in its center is aligned with the hole created in the bottom wall of the T-flask. Figure 1 shows the different parts of the proposed design.

#### 2.1.2 T-flask sterilization

After completing the fabrication step, the sterilization of T-flask was achieved by microwaving the vessel based on a study on reusing tissue culture vessels (Sanborn et al., 1982). In this method the T-flask was rinsed with DI water, it was then microwaved for 3 min using a 2.45 GHz home type microwave. A container including 200 ml water was placed in the microwave during the process to act as heat sink. LB broth medium was prepared by suspending 20 g of LB broth powder purchased from Thermo Fisher Scientific (Waltham, MA) in 1 L purified water. After a gentle mixing, the solution was autoclaved for 15 min pH was then adjusted to 7.2, and the medium was filtered. 10 ml of LB broth medium was added to the modified and sterilized T-flask to evaluate the efficacy of the sterilization method. The T-flask was then placed in an incubator set at 37°C and 5% CO_2_ and monitored for any sign of contamination over 7 days.

#### 2.1.3 Removing extractables and leachables

To attach the silicone membrane on the hole in the bottom wall of the T-flask, epoxy glue was used. The same type of glue was used for attaching the sampler to the silicone membrane. As a result of this application, the cells will be exposed to the glue in the setup during the cell culture process. Because of the toxic nature of the glue, it has the potential to adversely affect the cells cultured in the modified T-flask. Observations from cells cultured in modified T-flasks confirmed this by showing a sharp decline in cell viability after day 1. Different types of glue were examined, and cytotoxicity remained as the major problem for all trials. A method was developed to resolve the cytotoxicity issue. In this method, the modified and sterilized T-flasks were rinsed with 10 × solution of Phosphate Buffered Saline (PBS) purchased from Thermo Fisher Scientific (Waltham, MA). The rinsing process was conducted twice a day for 4 weeks. The efficacy of the method was studied by measuring the viability of cells cultured in T-flasks treated with different glues and comparing them with the viability of the cells cultured in the control T-flask. Control T-flask was a non-modified T-75 flask with no glue treatment. The T-75 flask treated with epoxy glue and rinsed with PBS over 4 weeks was the second group. The third group was a modified T-75 flask treated with “Cyberbond Apollo Ethyl Cyanoacrylate” glue without being rinsed, and a modified T-75 flask treated with Krazy glue without being rinsed was the fourth group. To evaluate the efficacy of the developed method, mouse-mouse hybridoma cells, SP2/0-Ag14 (ATCC, Manassas, VA) were cultured in different groups of T-flasks with a seeding density of 6.3 × 10^4^ viable cells/mL. The viability of the cells in each group was measured using the trypan blue dye technique and monitored for 7 days.

#### 2.1.4 Cell attachment on silicone membrane

The attachment of cells on the silicone membrane, when culturing adherent cells in the modified T-flask, is an important point that was studied. This is specifically important as the formation of a monolayer of cells on the silicone membrane increases the thickness of the layer. Because the measurements depend on the diffusion rate of the CO_2_ through silicone membrane, the attachment of cells to the silicone layer could affect the sensor measurements. Therefore, the cell attachment was investigated through DAPI staining (Invitrogen, Waltham, MA). For this purpose, sterilized pieces of silicone membrane (1 cm × 1 cm) were placed in 6-well-plates, covering the bottom of each well. Adherent Chinese hamster ovary (CHO-K1) cells were cultured in each well with a seeding density of 3.1 × 10^4^ cells/cm^2^. The cells were maintained in 3 ml of complete medium comprised of 10% v/v Fetal Bovine Serum (FBS) (ATCC, Manassas, VA), and 90% v/v of Dulbecco’s Modified Eagle’s Medium (DMEM) (ATCC, Manassas, VA). The plates were then shaken gently to distribute cells evenly in the wells. The silicone membranes were collected on days 1, 2, 3, 4, 7, and 8, fixed in 4% paraformaldehyde (TissuePro Technology, Gainesville, FL) and stored in PBS (Thermo Fisher Scientific, Waltham, MA). 5 mg/ml DAPI solution was prepared by dissolving the content of the vial in 2 ml of DI water. The prepared DAPI solution was then diluted in PBS to achieve the concentration of 300 nM. The membranes were then stained by adding 300 µl of the diluted DAPI staining solution to them. After being incubated for 5 min and rinsed with PBS for 3 times, the fluorescence microscope was used for imaging the stained silicone membranes.

#### 2.1.5 Biofouling on silicone membrane

In the proposed noninvasive method for CO_2_ measurement, dissolved CO_2_ in the cell culture medium is measured based on the initial diffusion rate of the CO_2_ through silicone membrane attached to the bottom wall of the T-flask. Therefore, it is important to investigate biofouling on the silicone membrane during cell culture processes and its potential effect on the sensor measurements. To address this, precalibration and postcalibration processes were conducted. Precalibration refers to the calibration conducted prior to culturing cells in the modified T-flask. During this process, 75 ml of DI water was added to a modified T-75 flask. The T-flask was then placed in a 5% CO_2_ incubator at 37°C, and the gas mixtures with different percentages of CO_2_ (0%, 2.5%, 5%, 7.5%, and 10%) were sparged in the DI water. The sensor reading was recorded for each percentage of CO_2_ sparged. The postcalibration refers to the calibration conducted after 7 days of culturing hybridoma cells in the modified T-flask. For this purpose, suspension mouse-mouse hybridoma cell line, SP2/0-Ag14 (ATCC, Manassas, VA) were cultured in a modified T-75 flask. 21 ml of complete medium (90% v/v of DMEM supplemented with 10% v/v of FBS) were added to the flask. The seeding density in T-flask was 5×10^4^ viable cells/mL. In day 8, cell culture medium was removed from T-flask, and after rinsing the T-flask with DI water, 75 ml of DI water was added to it. Similar to the precalibration process, different percentages of CO_2_ were sparged in the DI water. The measurements of sensor corresponding to each percentage (0%, 2.5%, 5%, 7.5%, and 10%) were then recorded. The results from both precalibration and postcalibration were compared.

#### 2.1.6 Optical measurement system

To measure DO and pH throughout the cell culture processes, the optical patch sensor system, developed by authors’ laboratory, was used (Bambot et al., 1994). The DO and pH patches were autoclaved at 120°C for 25 min. After removing the adhesive liners, the patches were attached to the bottom wall inside the T-flask using sterile forceps. The vessel was then placed on a reader which is in the shape of a disk.

### 2.2 Fermentation and cell culture

#### 2.2.1 *Escherichia coli* (*E. coli*) fermentation

BL21(DE3) *E. coli* was purchased from Invitrogen (Wal-tham, MA). 100 μl of the stock was added to 100 ml of LB broth in a 500 ml shake flask and allowed to grow at 37°C and 250 rpm for 20–24 h. The formulation of the medium was 10 g casein peptone, 5 g yeast extract, and 5 g sodium chloride per liter. The preculture was used to inoculate a modified T-25 flask culture. This bacterium is commonly grown in shake flasks for small-scale studies. However, in this experiment *E. coli* was grown in modified T-flasks as a proof-of-concept study. 10 μl of the batch was diluted in 10 ml of LB broth medium in the T-flask which resulted in the initial optical density (OD) of 0.495. 10 μl of Kanamycin was added to the T-flask to isolate the *E.coli*. During the experiment the shaking speed and incubating temperature were set at 75 rpm and 37°C, respectively. The bacterial culture was maintained for approximately 10 h. The OD of culture was measured at three time points at 600 nm (OD600).

#### 2.2.2 Yeast culture*-pichia pastoris*

X-33, *Pichia pastoris* Yeast strain was purchased from Invitrogen (Waltham, MA). 100 μl of *Pichia* stock was added to 100 ml of Yeast Extract Peptone Dextrose (YPD) medium in a 500 ml shake flask and allowed to grow at 37°C and 250 rpm for 20–24 h. YPD medium was prepared by suspending and dissolving 20 g of dextrose in 200 ml of deionized water (DI water). In a separate container, 10 g of yeast extract, and 20 g of peptone were then suspended in 800 ml of DI water and dissolved with gentle stirring and autoclaved for 15 min at 15 psi and 121°C. After the pH was adjusted to 6.5 ± 0.5 at 25°C, the autoclaved solution was added to the dextrose solution prepared previously and filtered. The preculture was then used to inoculate the T-175 flask culture equipped with noninvasive monitoring system for DCO_2_. The DO patch was attached to the bottom of the T-flask for online monitoring of the DO throughout the process. 200 ml YPD medium was added to the T-flask, and it was supplemented with 200 μl of Zeocin to select the *Pichia* transformants only. The shaking speed and temperature were set at 200 rpm and 27°C during the experiment.

#### 2.2.3 CHO-K1 cell culture

Adherent Chinese hamster ovary (CHO-K1) cells, purchased from ATCC (Manassas, VA), were thawed from liquid nitrogen tank and after a few passages were inoculated in a modified T-175 flask. The seeding density was 4.3 × 10^4^ cells/cm^2^. 53 ml of complete medium composed of 10% v/v Fetal Bovine Serum (FBS) (ATCC, Manassas, VA), and 90% v/v HAM’s F12 medium with L-Glutamine (Lonza, Walkersville, MD) was used as the final volume. The T-flask was then placed in a 5% CO_2_ incubator set at 37°C. DO and pH patches were attached inside the T-flask prior to adding cells.

#### 2.2.4 Mouse-mouse hybridoma cell culture

Suspension mouse-mouse hybridoma cell line, SP2/0-Ag14 (ATCC, Manassas, VA) were thawed from liquid nitrogen tank and after a few passages were transferred to modified T-75 flasks to study the effectiveness of the method developed for removing leachables and extractables. 21 ml of complete medium (90% v/v of DMEM supplemented with 10% v/v of FBS) were added to each flask. The seeding density in each T-flask was 6.3 × 10^4^ viable cells/mL. Cell density and viability were determined using hemocytometer and trypan blue dye (Lonza, Walkersville, MD) technique.

## 3 Results and discussion

### 3.1 Noninvasive measurement of DCO_2_

**FIGURE 2:**
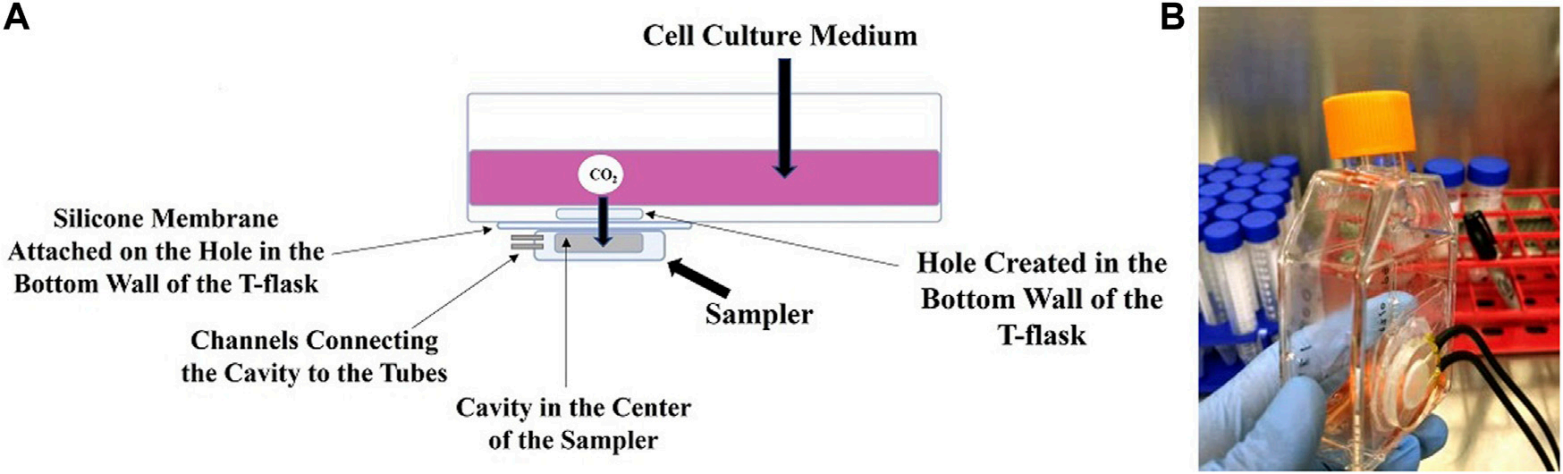
The schematic **(A)** and photograph **(B)** of the modified T-flask. The CO_2_ gas from the cell culture medium diffuses through the silicone membrane into the sampler, where it is collected and transferred to a CO_2_ sensor *via* black gas-impermeable tubing.

During the cell culture process in the modified T-flask, the CO_2_ dissolved in the culture medium diffuses through the silicone membrane. It is then collected in the sampler and transferred to the sensor for measurements. These steps are shown in Figure 2A. A modified T-flask equipped with noninvasive monitoring system for dissolved CO_2_ and ready to be connected to the sensor is shown in Figure 2B. The noninvasive nature of the sampling method in the proposed system resolves the issues associated with the original method. Minimizing the chance of contamination is the most important advantage of the method as submerging a sampling loop in the cell culture medium is no longer required. Furthermore, the proposed design resolves the issue regarding shear stress. As there is no need to place a sampling loop inside the cell culture medium, no unwanted shear stress is applied on the cells if shaking is needed. Additionally, the proposed method expands the application of the measurement method to cell culture processes with low working volume. The setup was evaluated by culturing different cell types in the modified T-flask. Results from bacteria, yeast and mammalian culture processes show that the DCO_2_ measurements from the proposed method corroborates the OD, DO and pH measurements obtained from the experiments. Therefore, the proposed method is an efficient way for measuring the CO_2_ dissolved in the cell culture medium in a noninvasive way.

#### 3.1.1 Calculation of initial diffusion rate

The rate-based method is based on correlating the CO_2_ concentration in the cell culture medium with the initial diffusion rate of the CO_2_ through the permeable silicone membrane. The mass balance equation for the system is written as:

$$V\frac{dC}{dt}=kA\left(C_{g}-C\right)$$ (1)

**FIGURE 3:**
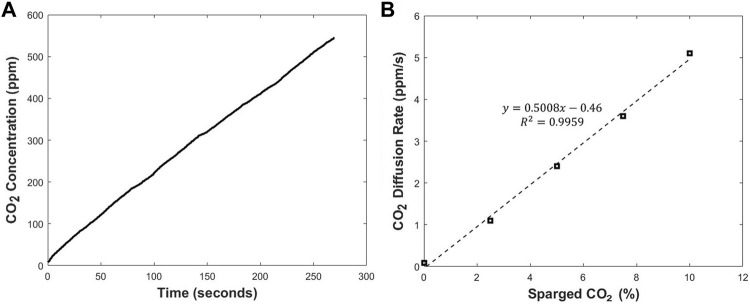
The CO_2_ concentration profile during a single recirculation period **(A)** and the relationship between the slope of CO_2_ concentration profile and the CO_2_ level in cell culture medium **(B)**.

Where V is the total volume of the system, C is the CO_2_ concentration in the sampling loop, t is time, k is the mass transfer coefficient, A is the total mass transfer area or the area of the silicone layer that is in contact with cell culture medium, and Cg is the CO_2_ concentration in the culture medium. In the current setup the mass transfer happens through the silicone membrane attached on the hole created in the bottom wall of the T-flask. Therefore, A in Eq. 1 refers to the surface area of the hole or the area of the silicone membrane covering the hole. Similar to the original design, the CO_2_ concentration in the medium is linearly proportional to the initial diffusion rate of the CO_2_ through the silicone membrane. Each measurement cycle starts with a purge period to remove the CO_2_ in the sampler. The purge period is followed by a recirculation period where the CO_2_ collected in the sampler is transferred to the sensor. After the start of recirculation period, the concentration of the CO_2_ in the sampler increases linearly with time over the first few minutes. This is presented in Figure 3A. The slope of the line depends on different parameters such as the total volume of the system, the surface area of the silicone membrane, and mass transfer resistance of the silicone membrane to the CO_2_. By changing any of these parameters, the slope would vary. The initial diffusion rate of CO_2_ through the silicone layer can be calculated by fitting the concentration profile in the first 2 min to a linear equation. By rearranging Equation 1, it can be concluded that the initial diffusion rate of CO_2_ through the silicone membrane is linearly proportional to the concentration of CO_2_ in the medium. This can be seen in Figure 3B. The CO_2_ diffusion rate across the silicone membrane is measured and recorded via LabVIEW (National Instruments) (Chatterjee et al., 2015).

#### 3.1.2 CO_2_ sensor calibration

The calibration processes were conducted in the condition similar to the actual cell culture experiments. This includes the cell culture medium, agitation speed, and temperature. For mammalian cell culture processes, the calibration process was conducted by adding complete medium (90% v/v of DMEM supplemented with 10% v/v of FBS) to the modified T-flask and placing the T-flask in a 5% CO_2_ incubator set at 37°C. Different percentages of CO_2_ (0.0%, 2.5%, 5.0%, 7.5%, and 10.0%) were then sparged in the cell culture medium. For bacterium and yeast cultures, different percentages of CO_2_ (0.0%, 2.5%, 5.0%, 7.5%, 10.0%, 12.5%, 15.0%, 17.5%, and 20.0%) were sparged in LB broth and YPD media. The desired gas mixtures with specific CO_2_ percentages were obtained by mixing pure N_2_ and CO_2_ through two mass flow controllers (Digital Pressure Controller, Single-Valve, 0–30 psia, Cole-Parmer, Vernon Hills, IL). The concentration of CO_2_ dissolved in the cell culture medium corresponding to each CO_2_ percentage that was sparged was calculated based on Henry’s law. The Henry’s law constants for oxygen and carbon dioxide at 27°C and 37°C were calculated based on the constants in Henry’s law constants compilation (Sander 2015). For each concentration of CO_2_ dissolved in the cell culture medium, the initial diffusion rate of CO_2_ through the silicone membrane was measured and recorded by LabVIEW software. The initial diffusion rate profile was converted to the dissolved CO_2_ profile in cell culture experiments using the data from the calibration processes.

#### 3.1.3 T-flask sterilization

The modified T-flask was sterilized based on the procedure described previously. The T-flask was then maintained in the 5% CO_2_ incubator and monitored over 7 days. During this process, no sign of contamination was observed. This procedure was repeated and proven to be effective even in longer periods of time, and in cell culture processes.

#### 3.1.4 Removing extractables and leachables

**FIGURE 4:**
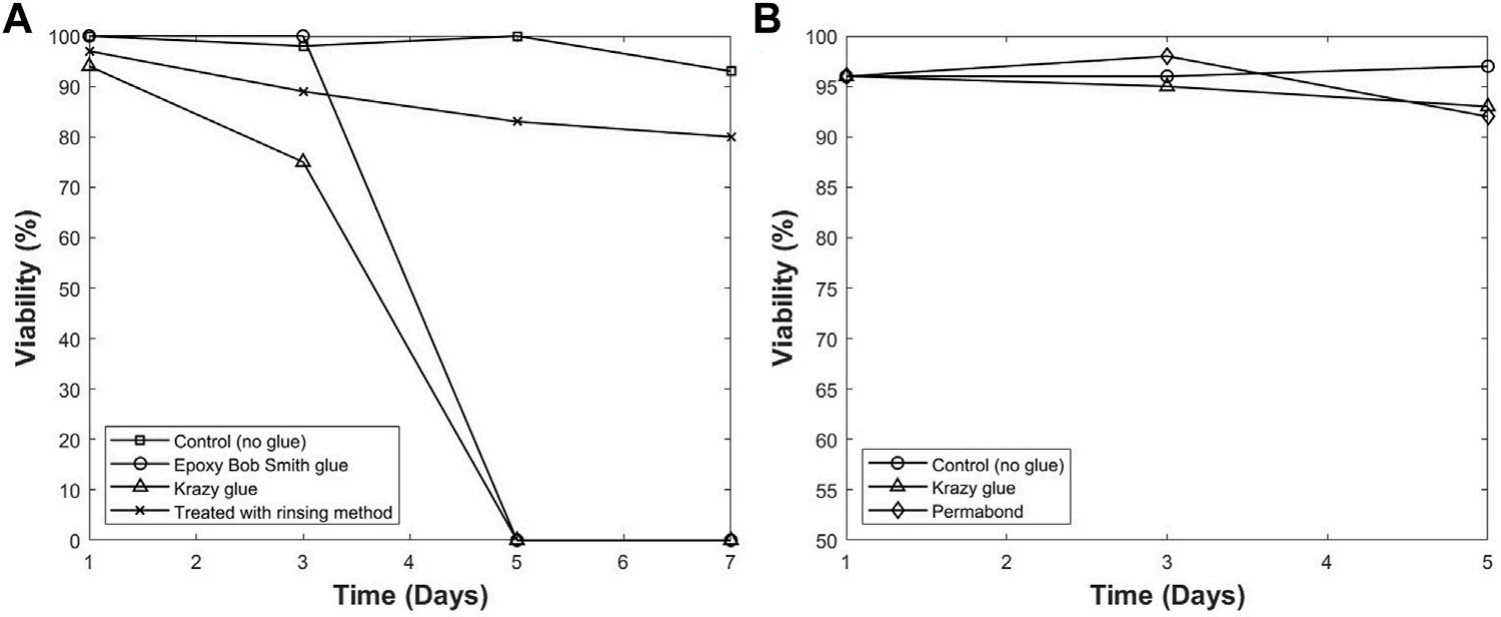
The viability of hybridoma cells cultured in different modified T-flasks with/without pre-rinsing **(A)**, and after optimized pre-rinsing procedure **(B)**. The high viability with pre-rinsing shows that the optimized pre-rinsing procedure is an effective way to remove the cytotoxicity of the adhesive.

A method to resolve the cytotoxicity issue was developed and described previously in this manuscript. Figure 4A shows the viability of cells in each T-flask over a period of 7 days. It was observed that cells cultured in the control T-flask maintained a high viability between 90% and 100%. Additionally, the T-flask treated with PBS rinsing method has a higher viability compared with other groups that were not rinsed. The results from this study show that the rinsing treatment was an effective method. Therefore, more studies were conducted to optimize the method to remove leachables and extractables within 3 days and increase the viability even further. The final procedure used for all T-flasks in this report is as follows: a T-75 flask was rinsed with PBS thoroughly, and approximately 30 ml PBS was added to the T flask. The vessel was then placed on a shaker in an incubator at 37°C, and the shaking speed was set at 100 rpm. After several hours, the PBS inside the T-flask was removed, and the T-flask was rinsed with PBS thoroughly. This step was conducted twice a day for 3 days. After each rinse, the T-flask was returned on the shaker and incubated at 37°C. The cell viability was monitored for 5 days in the T-flasks treated with this optimized rinsing method. Figure 4B shows the viability for cultures in different T-flasks treated with different glues after being rinsed by the optimized process. The results show that the developed process for removing leachables and extractables was successful. This method was used repeatedly in different experiments and high viability was observed in all trials. The method was successful even when DI water was used in replacement of PBS for rinsing process.

#### 3.1.5 Cell attachment on silicone membrane

**FIGURE 5:**
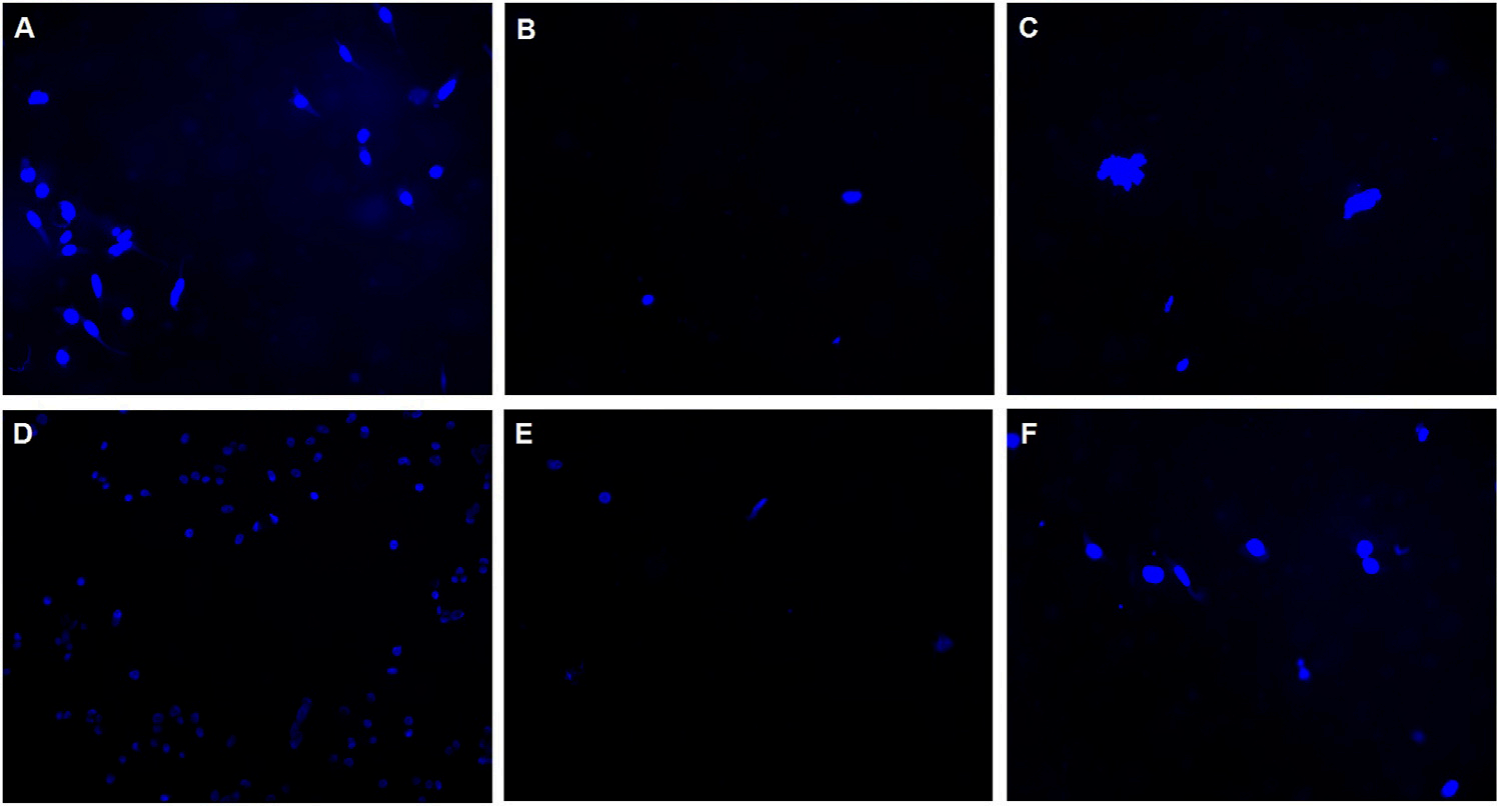
Silicone membranes cultured with CHO-K1 cells and stained with DAPI to investigate the cell attachment on the membrane. Images represent the distribution of cells attached on silicone membranes in **(A)** Day 1, **(B)** Day 2, **(C)** Day 3, **(D)** Day 4, **(E)** Day 7, and **(F)** Day 8.

To study the cell attachment on the silicone membrane, CHO cells were cultured on silicone membranes. Membranes were then stained with DAPI and prepared for imaging. Each image shown in Figure 5 represents the cell density on the silicon membrane for each day of culture. As it is observed in the images, the cell attachment on the silicone membrane is not significant. Therefore, the authors believe that the cell attachment does not affect the measurements via the silicone membrane.

#### 3.1.6 Biofouling on silicone membrane

**FIGURE 6:**
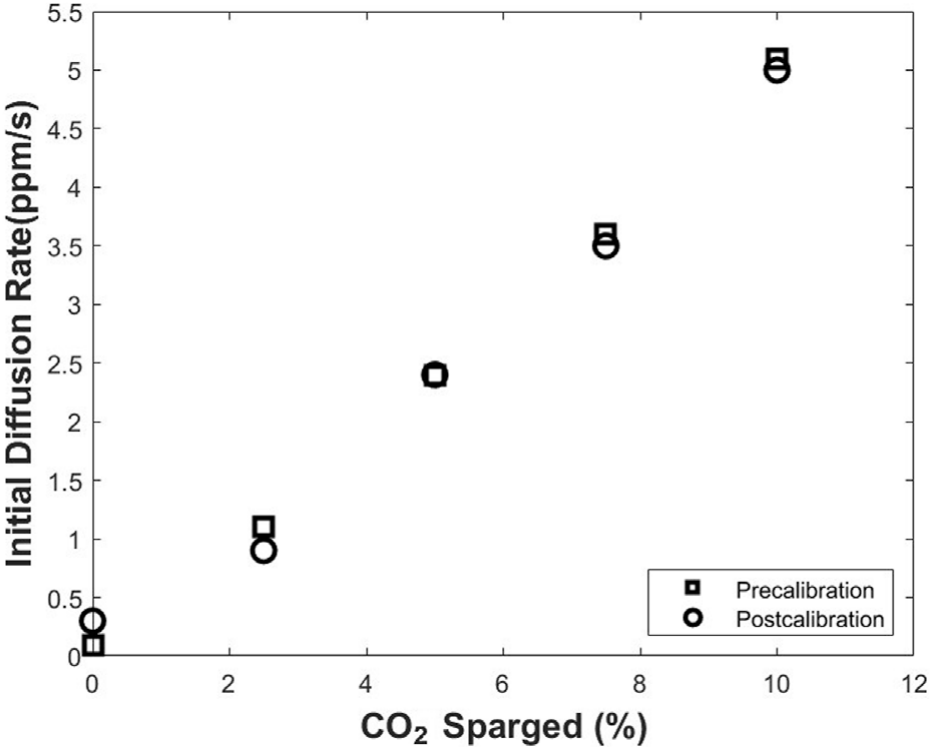
Pre- and post-calibration of the CO_2_ monitoring device before and after a 7-days cell culture. The comparison between the two data sets indicates no significant biofouling throughout the cell culture processes.

Based on the relation from Eq. 1, the thickness of the silicone membrane is a parameter affecting the sensor measurements. Therefore, biofouling on the silicone membrane could result in an increase in the thickness of the membrane and a decrease in the initial diffusion rate of CO_2_ through the silicone membrane. Calibration processes before the culture of cells in the modified T-flask (precalibration) and after 7 days of culture of cells in the T-flask (postcalibration) were conducted to study the biofouling and its effect on the initial diffusion rate of CO_2_ via the silicone membrane. The data from both processes are provided in Figure 6. A comparison between the two series of results indicates that the difference between the measured initial diffusion rate of CO_2_ in precalibration and postcalibration processes is negligible, and biofouling is not a concern in the long-term measurement processes.

### 3.2 Fermentation and cell culture

CO_2_ is involved in major metabolic pathways, and as one of the main extracellular metabolites, it could be used for estimating the metabolic state of cells. In addition, dissolved oxygen and pH are both indicators of cell growth and metabolism. In other words, increase in the cell density is associated with decrease in oxygen level as oxygen is consumed during the respiration. In addition, a decrease in the pH of the cell culture medium is an indication of the increase in the biomass because lactic acid is produced. Furthermore, DO and pH provide useful information about the start of the cell death as start of the death phase is accompanied with discontinuation of the oxygen consumption and acidification. (Lagadic-Gossmann et al., 2004; Yang and Balcarcel 2004; Naciri et al., 2008; Günter et al., 2018; Beal et al., 2020). During the yeast, and CHO cell culture processes, DO and pH values were monitored using the optical sensors. DCO_2_ was measured using the proposed noninvasive method. The details of the conditions for each experiment were described previously, and the results for each culture are discussed here:

#### 3.2.1 *Escherichia coli* (*E. coli*) fermentation

**FIGURE 7:**
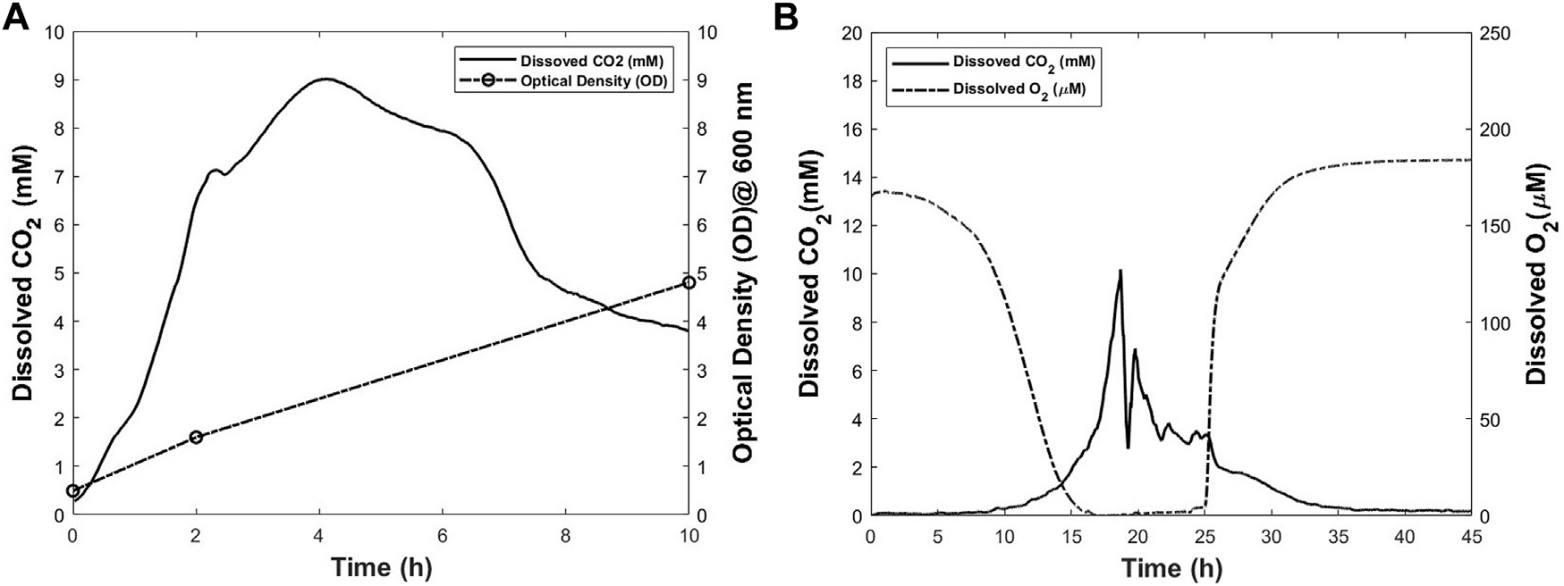
Dissolved CO_2_ and other process parameters for *Escherichia coli* **(A)** and *Pichia pastoris* **(B)** cultures. The dissolved CO_2_ profiles were obtained using the proposed method.

The process was started with the optical density (OD) of 0.495. Approximately 2 h after the start of the experiment, an increase in OD value from 0.495 to 1.6 was observed. The final OD measured for *E. coli* culture was 4.8. The increase in OD throughout the process is an indication of the growth of the cells. Different phases of the cell growth could be seen in the CO_2_ profile obtained from noninvasive measurements. The profile is shown in Figure 7A. In this experiment, the inoculum was large in size and young in age which usually results in a fast adjustment. This is reflected in the short lag phase which lasts less than 30 min. The lag phase is followed by the log phase lasting for approximately 1.5 h. During the log phase, the cells multiply rapidly, and produce CO_2_ with a high rate. This is observed by a rapid increase in the CO_2_ profile. In this phase, the cell mass increases exponentially. The OD of 1.6 measured at time point of 2 h is an indication of the rapid increase in cell mass. After 2 h from the start of culture, the log phase ends, and is followed by the deceleration phase which continues for approximately 2 h. At time point of 4 h after the start of cell culture process, the stationary phase starts. Figure 7A shows no clear distinction between the stationery and death phases. One possible reason could be the low volume of cell culture broth used in this experiment. This results in the early depletion of the nutrients as well as accumulation of toxic products in the cell culture medium. Therefore, the death phase starts before the stationary phase ends. Death phase starts in less than 6 h from the beginning of the culture and continues until 10 h after the start of the experiment. During this phase, the DCO_2_ in culture decreases accordingly until the end of the experiment where the measured OD is 4.8.

#### 3.2.2 Yeast culture*-pichia pastoris*

*Pichia* is commonly used in cell biology studies because of its similarities to animal cells in many aspects (Polymenis and Schmidt 1997; Iglinski-Rochester 2016; Litsios et al., 2019). Therefore, this experiment was conducted as proof-of-concept study where yeast was cultured in a modified T-175 flask. DO and DCO_2_ levels were monitored during the cell culture process and the corresponding profiles are shown in Figure 7B. The OD was measured at the start and end points of the culture. The increase in OD from 0.012 to 12.6 indicates the cell growth throughout the process. In the first 4 h of the culture no change is observed in the oxygen and CO_2_ level in the cell culture medium. At time point of 4 h, a decrease in oxygen level is observed. However, this decrease happens at low rate until the time point of 9 h from the start of the culture. At this time, the decrease in oxygen level starts to happen at a higher rate in comparison with the first 9 h of the culture. Interestingly, at the same time, the CO_2_ level starts increasing. This trend continues until approximately 19 h after the initiation of the culture, when a rapid decrease in CO_2_ is observed. The exact reason is not clear, but this could be because of the lack of sufficient nutrients and oxygen (Tervasmäki et al., 2018; Koutsokali and Valahas 2020). At about the time point of 16 h, dissolved oxygen level reaches zero. In other words, the cell culture medium is exhausted from oxygen at this time. No oxygen is sparged in the medium throughout the process and the oxygen level remains zero until the time point of 24 h. In addition, there is no clear distinction between the stationary phase and the death phase. Low volume of culture broth could be one reason. At time point of 24 h, CO_2_ level has decreased drastically. At this time, the increasing trend in oxygen dissolved in the cell culture medium starts until it reaches equilibrium with gas phase at time point of 30 h. During this period, a decrease in CO_2_ level is observed until the end of process where it reaches zero. It is worthwhile to mention that during the time ranges of 0–9 and 25–30 h, both DO, and DCO_2_ concomitantly change at rates lower than the rest of process. The results from this experiment prove that the proposed method for noninvasive measuring of DCO_2_ is an effective method to provide more detailed information from the process.

#### 3.2.3 CHO-K1 cell culture

**FIGURE 8:**
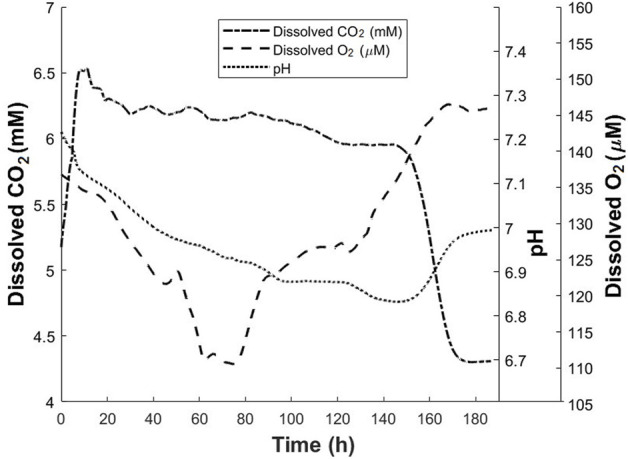
Dissolved CO_2_, dissolved oxygen and pH profiles for CHO-K1 culture in modified T-175 flask. The dissolved CO_2_ profile was obtained using the proposed method.

CHO-K1 cells were cultured in a modified T-175 flask. DO, DCO_2_ and pH values were monitored throughout the process and plotted in Figure 8. CHO-K1 cells were thawed and grown in T-175 flask, and after a few passages, the cells were added to a modified T-175 flask with a seeding density of 2.8 × 10^4^ cells/cm^2^. 53 ml of complete medium was the final volume of the culture. The incubator was set at 5% CO_2_ and 37°C. Based on the calculations, the dissolved CO_2_ in the cell culture medium in an 5% CO_2_ incubator at 37°C is approximately 4.5 mM. For the first 5 h of the culture, an increase in the CO_2_ level is observed until the CO_2_ dissolved in the cell culture medium reaches 5.9 mM. As the cells grow, the increase in the CO_2_ level as well as the lactic acid produced by the cells, result in the decrease in pH. Additionally, oxygen is consumed during the cell growth. Therefore, a decrease in pH and DO levels is observed during this time. A sharp increase in CO_2_ to 6.5 mM is observed until time point of approximately 8 h. Authors believe that this sudden increase in CO_2_ level between 5 and 8 h is a result of the fluctuation in the CO_2_ released by incubator and this affects the readings until approximately 20 h after the start of the culture when CO_2_ reaches 6.3 mM. During this time, as the cells are growing, a slight decrease in DO and pH is observed. After approximately 20 h of the start of the culture, the increase in the CO_2_ ends, and a slight decrease in CO_2_ level happens over the next 8 h when it reaches 6.2 mM. After this time point, between 28 and 82 h of the culture, CO_2_ level remains stable at approximately 6.2 mM with a slight periodic change. At time point of 20 h, DO starts decreasing at higher rate than before; therefore, between time points of the 20–45 h, DO decreases from 134 to 122 μM, a change of about 12 µM. This indicates higher rate in oxygen consumption due to the increase in biomass. During this time, the pH decreases slightly, by 0.1 points due to the lactic acid and CO_2_ produced by cell metabolism. Between the time ranges of 52–63 h, the DO level decreases at a rate even higher than previous rate until it reaches 111 µM. The pH changes very slightly in this time range. For approximately next 15 h, the DO level remains constant at 111 µM showing that the oxygen uptake rate is equal to the oxygen consumption rate. The CO_2_ level remains almost constant at approximately 6.2 mM during this period while the pH changes slightly by about 0.05 points. In other words, during the time range of 63–78 h, the slightest changes in DO, DCO_2_ and pH are observed. At time point of approximately 80 h, the DO level starts increasing and this continues until the time point of 130 h. This means that cells are consuming oxygen at a lower rate compared with before. During the same time, a gradual decrease in the CO_2_ level is observed. One reason for this decreasing trend is the decrease in cell growth which results in CO_2_ production at a lower rate. The pH value continues decreasing by 0.1 points during this time period. From 130 to 150 h, the DO increases at a higher rate, the CO_2_ level remains almost constant, and the pH decreases at a rate higher than the rest of process. The accumulation of lactic acid in the cell culture medium could be one reason for the larger change in the pH value during this period. At 150 h of the culture, the pH starts increasing, the DCO_2_ level starts decreasing, and the DO continues increasing, all at a high rate. This trend continues until all three parameters of DO, pH and DCO_2_ concomitantly reach an approximately constant level at time point of 170 h. The measurements were continued until 185 h from the start of the experiment. During the time period of 170–185 h, DO, pH and DCO_2_ are constant. At point of 185 h, the experiment was terminated, and the measured final density was 7.2 × 10^5^ cells/cm^2^. In addition, the viability was 84%. In the time range of 170–185 h, DCO_2_ level is constant at 4.3 mM. Based on the calibration conducted for this study, this concentration is approximately the concentration of dissolved CO_2_ in the cell culture medium when 5% CO_2_ is present in gas phase. In other words, the CO_2_ measured during this period is resulted from the CO_2_ inside the incubator. This could be interpreted that during this time no additional CO_2_ is produced by the cells. Interestingly, in the same period, the DO and pH values remain at constant levels of 145 μM and 7.0 points respectively. This concomitant lack of change in all three parameters is an indication of the lack of significant metabolic activity (Günter 2018; Naciri et al., 2008). As it can be seen, the data obtained from the proposed noninvasive method provides more details about the cell culture process. This data is obtained without any direct contact of sensor with the cell culture environment and is complementary to the other data. This additional information helps to gain a better understanding of the cell culture process while no adverse effect is caused by the measurement method.

## 4 Conclusion

Small-scale cell culture studies play crucial role in advancing knowledge about cells and cell culture processes by helping in developing small-scale models, and mitigating problems at early stages of bioprocesses. Therefore, it is imperative to provide more quantitative data about important parameters in the cell culture environment such as dissolved CO_2_ (DCO_2_). Monitoring CO_2_ dissolved in the cell culture medium will help to provide more reliable information about culture conditions such as alterations in metabolism and contamination. In addition, online measurement of DCO_2_ would help in maintaining its level in cell culture medium within the desired range. This is specifically important as studies show that high levels of CO_2_ could have adverse effects on cell growth and protein production (Blombach and Takors 2015; Jagschies et al., 2018). Different electrochemical and optical sensors are developed to monitor this analyte. However, these methods are associated with significant issues such as being bulky, requiring recalibration, being prone to drift, not having enough durability, and causing cytotoxicity. In order to resolve the aforementioned problems, a rate-based method was developed and validated by authors’ lab, and the results were reported previously. The method works based on submerging a sampling loop in the cell culture medium and measures the dissolved CO_2_ concentration based on the initial diffusion rate of the CO_2_ through a silicone sampling loop. Despite the efficacy of the rate-based method, it is associated with inevitable drawbacks such as increasing the risk of contamination. Because the measurement method requires the presence of the sampling loop in the cell culture environment. In fact, most of other available methods for CO_2_ measurement are associated with risk of contamination. Reducing the risk of contamination is one of the principles in cell culture process, and it is specifically important in cell therapies where cells cannot be terminally sterilized. Therefore, in such cell culture processes, it is critical to reduce the number of open processes and the operational errors to eliminate the risk of contamination (Barone et al., 2020; Abou-el-Enein et al., 2021). This disadvantage is one main issue that must be addressed. Additionally, the presence of the sampling loop causes shear stress. This is not desirable in cell culture processes such as stem cell cultures where the destiny of cells could change depending on the shear stress from sampling loop while shaking or mixing is applied. The other disadvantage of the CO_2_ measurement via a sampling loop is that the measurement method is not practical for cell culture practices with low working volumes because the sampling loop must be fully submerged in the cell culture medium during the measurements. To address all the aforementioned disadvantages, we present a noninvasive method to measure the DCO_2_ in small-scale cell culture processes. Unlike the original design, in this method, the CO_2_ dissolved in the cell culture medium is measured without any direct contact with the cell culture medium. In the proposed noninvasive method, a small area of the vessel is replaced with a silicone layer which is permeable to CO_2_. Throughout the cell culture process, the CO_2_ diffuses through silicone membrane attached to the bottom wall of the T-flask. It is then collected in a sampler attached on the silicone membrane from outside. Therefore, in this method, sampling is conducted from outside of the cell culture vessel. Different cell culture processes such as *E.coli, Pichia*, and CHO cultures were conducted in the modified T-flask equipped with the noninvasive monitoring system. The CO_2_ measurements obtained from the proposed method were described and justified by other data obtained from the cell culture processes such as OD, DO and pH profiles. The results from these studies indicate that the CO_2_ from cell culture environment was successfully monitored in a noninvasive way. Therefore, the proposed prototype, featuring a sampler mounted outside the T-flask, is a promising method to provide detailed and reliable information about the cell culture environment, and has the potential to be integrated with other small-scale cell culture vessels.

## Data Availability

The original contributions presented in the study are included in the article/Supplementary Material, further inquiries can be directed to the corresponding author.
